# The Th17/Treg Immune Imbalance in Ulcerative Colitis Disease in a Chinese Han Population

**DOI:** 10.1155/2016/7089137

**Published:** 2016-02-09

**Authors:** Yang Gong, Yifan Lin, Ning Zhao, Xiaojuan He, Aiping Lu, Wei Wei, Miao Jiang

**Affiliations:** ^1^The General Hospital of Shenyang Military Region, Shenyang, Liaoning 110840, China; ^2^Institute of Basic Research in Clinical Medicine, China Academy of Chinese Medical Sciences, Beijing 100700, China; ^3^Beijing Wangjing Hospital, Beijing University of Chinese Medicine, Beijing 100102, China; ^4^University of Hawaii Cancer Center, Honolulu, HI 96813, USA

## Abstract

*Objective*. To investigate the Th17/Treg immune balance in the ulcerative colitis (UC) patients in a Chinese Han population.* Methods*. Ninety UC patients and 30 healthy subjects were enrolled. The serum IL-17 and TGF-*β*1 levels of these participants were measured with ELISA; the percentage of Th17 and Treg cells in peripheral blood was determined with flow cytometry.* Results*. In UC patients, the levels of IL-17 and Th17 were significantly higher compared with healthy subjects; the percentage of Th17 and IL-17 level in moderate and severe subgroup was significantly higher than in mild subgroup; a positive correlation existed between these two indexes and clinical activity index and endoscopic evaluation. TGF-*β*1 level and Treg cells in UC patients were lower than healthy subjects. TGF-*β*1 level in moderate and severe subgroup was lower than in mild subgroup. There was a negative linear correlation between Treg cells and clinical activity index, endoscopic evaluation. A positive correlation was detected between Treg cells and TGF-*β*1 level.* Conclusions*. Th17/Treg immune imbalance might play a crucial role in the development of UC. To induce the production of Treg cells and TGF-*β*1, inhibit the level of Th17 and IL-17, and thus recover the Th17/Treg immune balance might imply new therapeutic targets in UC management.

## 1. Introduction

Ulcerative colitis (UC), as one of the major inflammatory bowel diseases (IBD), is a debilitating disorder of the gastrointestinal tract, characterized by a dysregulated immune response to unknown environmental triggers, including the inflammation of the intestine mucosa and submucosa, with a group of clinical symptoms of diarrhea, mucus purulent blood stool, and abdominal pain [1, 2]. Currently UC is incurable, yet the symptoms can be managed through anti-inflammatory steroids or immunosuppressants [3]. UC prevalence is currently highest in Europe (505 per 100,000 persons) and North America (249 per 100,000 persons) [4]. Now the global prevalence is rising, including Asian area [5], with rapid increases in incidence rates in younger people, placing an increased strain on healthcare resources; thus it represents a significant global health burden that is of growing concern [6].

Recent progresses in the immune mechanisms implicated in chronic inflammatory disorders have led to a more in-depth knowledge of the pathogenesis of UC; some common T cell mediated mechanisms for inflammation—Th17/Treg immune balance—are recognized as crucial mediators of the tissue damage causing mucosal ulceration of the colon in ulcerative colitis, yet the pathogenic role of this specific immune balance is still controversial [7].

Th17 and Treg cells are both originated from CD4+ T cells, circulating in peripheral blood or in the spleen. Th17 (CD4+ IL-17+) cells, as one of the inflammatory helper T cells and an important proinflammatory cells, promote inflammation progressing with specific secretion of IL-17 [8]. Treg (CD4+ CD25+ Foxp3+) cells mainly secrete some cytokines such as transforming growth factor-beta (transforming growth factor-beta, TGF-*β*) and act as suppressor cells with unique immune regulation [9]. The normality of Treg cells plays crucial role in the maintenance of the body's immune tolerance, Treg cells accomplish their biofunctions via the inhibiting cytokines [10], which are important in the production and the proliferation of Treg cells and their mediation of immune tolerance [11].

UC is regarded as a kind of chronic inflammatory disease, under this condition, Th17 increases and Treg cell decreases. In our previous study, it was proved that the levels of IL-17 and Th17 in serum and colon of UC patients were significantly higher, yet the TGF-*β*1 and Treg cells were much lower compared with healthy control subjects [12]. Resveratrol in low dose (50 mg/kg·d) was reported to be able to regulate the Th17/Treg balance mainly through reducing the number of Th17 cells, and resveratrol in high dose (100 mg/kg·d) regulates this balance through both downregulating the number of Th17 and upregulating the number of Treg cells, thus to improve the inflammation in UC [13]. However, in some other studies, different conclusions were yielded; it was reported that only in moderate and severe UC patients, IL-17 could be determined, and the inflammation level was positively correlated with the level of IL-17 [14]. The Treg cells function was regarded as normal in the peripheral blood of UC patients; the number of Treg cells decreased during disease active stage, yet it increased during the disease remission period; the expression of Treg cells was higher in inflammation locations in mucous membrane of colon compared with noninflammation locations, yet still lower than control group [15].

Given these controversial studies, the variation of Th17/Treg immune balance in UC patients was still unclear. Therefore we conducted this clinical study to further investigate the change of Th17/Treg immune balance in a Chinese Han population with UC as well as the potential underlying mechanism.

## 2. Materials and Methods

### 2.1. Patients

**Figure 1:**
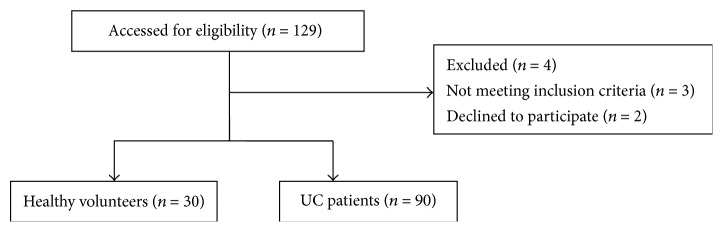
Study flow through study.

Ninety Chinese Han patients with UC were recruited from the General Hospital of Shenyang Military Region from July, 2010, to February, 2014. Thirty healthy volunteers were enrolled from the neighbouring community. The diagnosis of UC was made based on clinical, laboratory, endoscopic, and histologic examinations with reference to the suggested guidelines for the diagnosis and treatment of IBD, which were approved in China in 2007 by the Chinese Medical Association Society of Digestive Disease Branch of inflammatory bowel disease Collaborative Group [16]. The study flow chart is shown in Figure 1.

All UC patients enrolled were aged between 18 and 70 years, diagnosed with UC, the initial onset, chronic persistent, or chronic relapsing type. Diagnostic facilities for high-quality endoscopy, radiology, and pathology should be available. Priority was given to the mild and moderate UC patients; cases with severe disease would also be enrolled if they did not need emergency therapy.

None of the enrolled subjects had any serious complications, such as local stricture, intestinal obstruction, intestinal perforation, rectum polyp, toxic colonic dilatation, colon or rectum cancer, anus diseases, or any severe primary diseases in cardiovascular, cerebrovascular, liver, kidney, or hematopoietic system, or mental diseases. Women who were pregnant or preparing to be pregnant or lactating were excluded from the study.

The study was approved by the Ethics Committee of Beijing Dongzhimen Hospital (Approved number ECPJ-DBY-2010-013), in accordance with the World Medical Association Declaration of Helsinki. All participants signed written, informed consent before participating.

### 2.2. Sample Collection and Preparation

Peripheral venous blood (8 milliliters in each subject) was collected from each participant. The subjects had to be fasted for at least 12 hours before blood sample collection. The blood sample was kept in the room temperature for 1 h and then centrifugated for 10 minutes at 4°C, 3000 r/min. Supernatant was then frozen at −80°C. Information on gender, age, height, body weight, marital status, disease course, complications, medication, clinical activity index, and endoscopic evaluation were collected from each patient. Clinical activity index and endoscopic evaluation are determined with reference to the Chinese Medical Association Society of Digestive Disease Branch of inflammatory bowel disease Collaborative Group [16].

### 2.3. Enzyme-Linked Immunosorbent Assay (ELISA)

ELISA assay was performed according to the manufacturer's instructions of the ELISA kits (RapidBio, USA) for IL-17 and TGF-*β*1. The OD value at 540 nm was measured. The concentrations of IL-17 and TGF-*β*1 were calculated according to the standard curve. The Varioskan Flash was purchased from Thermo Scientific, USA.

### 2.4. Flow Cytometric Analysis

For detecting the percentage of Th17 cells, the PBMCs were stimulated with 20 ng/mL phorbol 12-myristate-13-acetate and 1 *μ*g/mL ionomycin in the presence of 2 mmol/mL monensin (Sigma-Aldrich, USA) in 24-well plates. After being stimulated for 4 hours (37°C, 5% CO_2_), the cells were collected and washed once with PBS. The cells were then incubated with APC-CD3 antibody and PE-Cy5-CD4 antibody at 4°C for 30 minutes. Next, the cells were fixed and permeabilized and stained with anti-human PE-IL-17 antibody at 37°C for 25 minutes. For detecting the percentage of Treg cells, the PBMCs were washed in PBS. Then the cells were stained with APC-CD3, PE-cy5-CD4, PE-Cy7-CD8, and FITC-CD25 antibodies at 4°C for 30 minutes. Then the cells were incubated with PE-Foxp3 antibody after fixation and permeabilization according to the manufacturer's instruction. All stained cells were analyzed by flow cytometry (FACSCalibur) and FlowJo software (Tristar, USA). The forward angle scattering light (FSC) and side scattering light (SSC) were adjusted to select the lymphocytes. Different cell subsets were detected by different cell labeling and gating. CellQuest software was used for data analysis and the percentage of positive cells was recorded.

### 2.5. Statistical Analysis

All data were analyzed by SPSS 17.0 software. All data except demographic data were expressed as mean ± SD. Measurement data in normal distribution were analyzed using Student's *t* test; variables deviated from normality were analyzed by nonparametric tests Wilcoxon rank sum test. Numeration data were analyzed using Chi-square analysis. Pearson linear correlation analysis was adopted for variables in normal distribution; otherwise Spearman rank correlation analysis was used. A *P* value of less than 0.05 was considered statistically significant.

## 3. Results

### 3.1. Demographic Features and Clinical Characteristics

Among 90 UC patients, 51 (56.7%) were male, the age ranged from 29 to 64 yr (37.19 ± 8.50); in 30 healthy control subjects, 17 (56.7%) were male and the age ranged from 28 to 61 (36.30 ± 9.12); there were no significant differences between the two groups in gender and age distribution (*P* > 0.05) as shown in Table 1.

In UC patients, 46 cases (51.1%) were diagnosed as mild type, 38 (42.2%) as middle type, and 6 (6.7%) as severe type. The clinical disease activity index was 6.80 ± 2.31; endoscopic index was 5.16 ± 1.99 (mean ± SD).

### 3.2. Serum IL-17 and TGF-*β*1 Levels in UC Patients and Healthy Subjects

**Figure 2:**
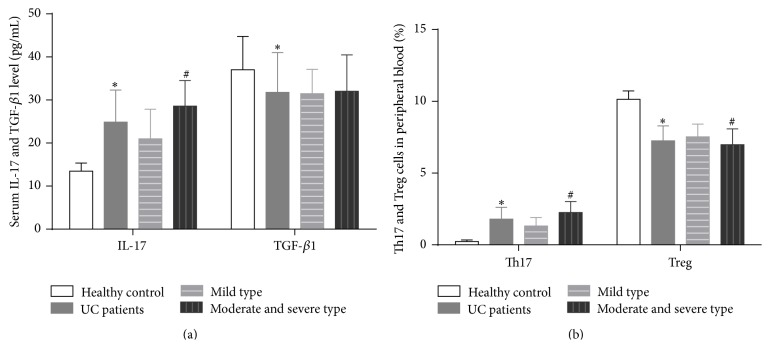
Serum IL-17 and TGF-*β*1 level (pg/mL) and percentage of Th17 and Treg cells in peripheral blood (%) in healthy control subject, UC patients, mild type, and moderate and severe subgroup. (a) Serum IL-17 and TGF-*β*1 level (pg/mL) in healthy control subject, UC patients, mild type, and moderate and severe subgroup. (b) Percentage of Th17 and Treg cells in peripheral blood (%) in healthy control subject, UC patients, mild type, and moderate and severe subgroup. *∗* presents *P* < 0.05 between UC patients and healthy control subject; # stands for *P* < 0.05 between mild type and moderate and severe type subgroups.

In UC patients, the serum IL-17 level was significantly increased and the TGF-*β*1 level decreased compared with healthy subjects (*P* < 0.05) as shown in Table 1 and Figure 2.

The UC patients were further classified into 2 subgroups based on their disease severity, mild type and moderate and severe type (only 6 patients were diagnosed as severe type; thus they were combined with the moderate type). The serum IL-17 level in moderate and severe type of UC patients was significantly higher than mild type of patients (*P* < 0.05), yet TGF-*β*1 showed no difference between the two subgroups as shown in Table 2.

### 3.3. The Percentage of Th17 and Treg Cells in Peripheral Blood

In peripheral blood of UC patients, the percentage of Th17 increased significantly and Treg cells decreased significantly compared with healthy subjects (*P* < 0.05) as shown in Table 1 and Figure 1.

**Figure 3:**
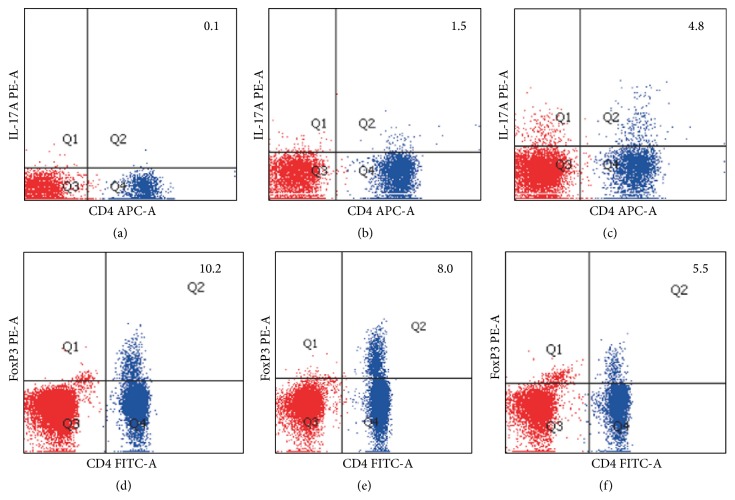
Flow cytometric analysis of the percentage of Th17 and Treg cells in peripheral blood. (a) Percentage of Th17 in peripheral blood in healthy control subjects. (b) Percentage of Th17 in peripheral blood in mild type subgroup of UC patients. (c) Percentage of Th17 in peripheral blood in moderate and severe type subgroup of UC patients. (d) Percentage of Treg cells in peripheral blood in healthy control subjects. (e) Percentage of Treg cells in peripheral blood in mild type subgroup of UC patients. (f) Percentage of Treg cells in peripheral blood in moderate and severe type subgroup of UC patients. In (a), (b), and (c), in the quadrant 1 (Q1), the IL-17A PE-A antibody is positive; the CD4 APC-A antibody is negative; in Q2, both the IL-17A PE-A and CD4 APC-A antibody are positive; in Q3, both the IL-17A PE-A and CD4 APC-A antibody are negative; in Q4, the IL-17A PE-A antibody is negative; the CD4 APC-A antibody is negative. In (d), (e), and (f), in Q1, the foxP3 PE-A antibody is positive; the CD4 FITC-A antibody is negative; in Q2, both the foxP3 PE-A and CD4 FITC-A antibody are positive; in Q3, both the foxP3 PE-A and CD4 FITC-A antibody are negative; in Q4, the foxP3 PE-A antibody is negative; the CD4 FITC-A antibody is negative.

In the moderate and severe subgroups, the percentage of Th17 was higher and the percentage of Treg cells was lower compared with mild subgroup (*P* < 0.05) as shown in Table 2 and Figure 3.

### 3.4. Correlation Analysis

In correlation analysis, it was detected that there was a positive linear correlation between serum IL-17 level and clinical activity index (*P* < 0.05, *r* > 0); between serum IL-17 level and endoscopic evaluation (*P* < 0.05, *r* > 0); between the percentage of Th17 in peripheral blood and clinical activity index (*P* < 0.05, *r* > 0); between the percentage of Th17 in peripheral blood and endoscopic evaluation (*P* < 0.05, *r* > 0); between the percentage of Th17 in peripheral blood and serum IL-17 level of UC patients(*P* < 0.05, *r* > 0); between the percentage of Treg cells in peripheral blood and serum TGF-*β*1 level of UC patients (*P* < 0.05, *r* > 0).

A negative linear correlation was detected between the percentage of Th17 in peripheral blood and clinical activity index (*P* < 0.05, *r* < 0) and between the percentage of Treg cells in peripheral blood and endoscopic evaluation (*P* < 0.05, *r* < 0). The correlation coefficient (*r*) and correlated *P* value of the analysis were shown in Table 3.

## 4. Discussion 

In this study, we verified the changing trends of serum Th17, Treg cells, IL-17, and TGF-*β*1 in UC patients, as well as their changes in patients with different disease severity and the correlation between the levels of these indexes and disease activity, thus to provide further evidence for the mechanism analysis in UC development.

UC is a chronic inflammatory bowel disorder with multifactorial pathogenesis factors. It has been proved that Th1/Th2 are involved in the development of UC [17, 18]; currently more and more evidence indicated that Th17/Treg balance is another key factor in UC.

Th17 and Treg cells are produced by CD4+ T cells and exist in spleen and peripheral blood in healthy individuals. Th17 and Treg cells are closely related in the process of differentiation and transformation, while they also can be independent or unified in the body's immune response which forms an immune balance as a switch of a variety of autoimmune diseases. In short, Th17 is one of the immune promoting cells, while Treg cell is a kind of immune suppressing cell; thus, the two kinds of cells and their balance are closely related to the immune function.

In UC patients, the increase of Th17 cell caused the higher level of serum IL-17, and the low Treg cell resulted in the decrease of serum TGF-*β*1; as a result, the autoreactive T cell was activated and inhibitive immune cytokines decreased, thus to aggravate the inflammation in the mucous membrane of colon. These results support that the Th17/Treg immune imbalance might play a crucial role in the development of UC. Thus to induce the production of Treg cells and TGF-*β*1, inhibit the level of Th17 and IL-17, and recover the Th17/Treg immune balance might imply new therapeutic targets in UC management.

Since Hovhannisyan et al. isolated Th17 and Treg cells from intestinal lamina propria of patients with IBD, the role of Th17 and Treg cells in pathogenesis of UC began to attract researcher's attention [19]. Then Ogino et al. proved that Treg cells were capable of suppressing colonic inflammation by downregulating Th17 responses via TGF-*β* by using UC mice model treated with Treg cells [20], which indicated the immune balance effect of Th17 and Treg cells. Yet, the pathogenesis of UC is still contradictive concerning some details, such as the changing trend of serum Th17, IL-17, Treg cells, and TGF-*β* in UC patients and their correlation with disease activity and severity.

In this study, we indicated that the levels of serum IL-17 and percentage of Th17 in peripheral blood of UC patients increased significantly compared with healthy control; and these two indexes were positively correlated with clinical activity index and endoscopic index, which improved that serum IL-17 and percentage of Th17 in peripheral blood increased as disease condition aggravated. Percentage of Th17 was positively correlated with serum IL-17 level, which supports the point that Th17 and IL17, which is secreted by Th17 cells, can promote the inflammation during the development of UC and keep increasing as disease aggravates; thus they might be used as markers for judging the UC disease severity. These results are similar to most previous studies [12, 13, 21].

We also found out that the serum TGF-*β* level and percentage of Treg cells in peripheral blood of UC patients were significantly lower than healthy individuals, and they showed negative correlation with clinical activity index and endoscopic index, which demonstrated that when UC disease aggravated, percentage of Treg cells in peripheral blood decreased, yet it is still unclear about the correlation between TGF-*β* and disease severity. Percentage of Treg cells was positively correlated with serum TGF-*β* level; Treg cells might be another important origination of TGF-*β*. Therefore, our study implied that Treg cells in the peripheral blood play the role of immunosuppression cells and should inhibit the inflammation in UC procedure. TGF-*β*, secreted by Treg cells, also possesses immunosuppression effect; thus, in the population who produce less Treg cells, there will be a higher incident rate of UC. Consequently, Treg cells and TGF-*β* transfer therapy is expected to be efficacious for UC and inhibit the immunosuppression in UC patients.

There are still some limitation in this study. The sample size in the severe type subgroup of UC patients was small so that we could only combine this subgroup with the moderate type subgroup in analysis. Thus more studies, especially clinical studies with larger sample size, are still warranted to explore the underlying mechanism of Th17/Treg immune balance in the development of UC.

## 5. Conclusions

In UC patients, the increase of Th17 cell caused the higher level of serum IL-17, and the low Treg cell resulted in the decrease of serum TGF-*β*1; as a result, the autoreactive T cell was activated and inhibitive immune cytokines decreased, thus to aggravate the inflammation in the mucous membrane of colon. These results support that the Th17/Treg immune imbalance might play a crucial role in the development of UC. Thus to induce the production of Treg cells and TGF-*β*1, inhibit the level of Th17 and IL-17, and recover the Th17/Treg immune balance might imply new therapeutic targets in UC management.

## Figures and Tables

**Table 1:** Demographic details and clinical characteristics of UC patients and healthy subjects.

	UC group	Control group	*P* value
	(*n* = 90)	(*n* = 30)	
Male sex, number (%)	51 (56.7)	17 (56.7)	1.000
Age, year	37.19 ± 8.50	36.30 ± 9.12	0.889
Clinical activity index	6.80 ± 2.31	—	—
Endoscopic index	5.16 ± 1.99	—	—
IL-17 (pg/mL)	24.86 ± 7.44	13.49 ± 1.87	<0.001^*∗*^
TGF-*β*1 (pg/mL)	31.77 ± 9.27	37.01 ± 7.75	0.002^*∗*^
Th17 (%)	1.80 ± 0.82	0.23 ± 0.12	<0.001^*∗*^
Treg (%)	7.25 ± 1.04	10.14 ± 0.59	<0.001^*∗*^

The numbers are shown as mean ± SD.

**Table 2:** Demographic details and clinical characteristics in mild type and moderate and severe type of UC patients.

	Mild type	Moderate and severe type	*P* value
	(*n* = 44)	(*n* = 46)	
Male sex, number (%)	29 (65.91%)	22 (47.83%)	0.064
Age, year	38.75 ± 9.76	35.6957 ± 6.87	0.207
Clinical activity index	5.18 ± 1.45	8.35 ± 1.88	<0.001^*∗*^
Endoscopic index	4.14 ± 1.03	6.13 ± 2.21	<0.001^*∗*^
IL-17 (pg/mL)	20.98 ± 6.88	28.58 ± 5.95	<0.001^*∗*^
TGF-*β*1 (pg/mL)	31.49 ± 5.63	32.04 ± 8.45	0.678
Th17 (%)	1.32 ± 0.59	2.26 ± 0.76	<0.001^*∗*^
Treg (%)	7.53 ± 0.89	6.98 ± 1.10	0.006^*∗*^

The numbers are shown as mean ± SD. *∗* stands for *P* < 0.05.

**Table 3:** Correlation analysis between the indexes in UC patients.

	IL-17	TGF-*β*1	Th17	Treg
Clinical activity index	0.498 (0.000)^△^	−0.032 (0.762)	0.653 (0.000)^△^	−0.404 (0.000)^△^
Endoscopic index	0.448 (0.000)^△^	−0.068 (0.379)	0.488 (0.000)^△^	−0.419 (0.000)^△^
IL-17	—	—	0.562 (0.000)^△^	
TGF-*β*1	—	—		0.209 (0.004^*∗*^)

The numbers are shown as *r* (*P*). ^*∗*^ *P* < 0.05. The correlation coefficient *r* > 0 shows positive correlation; *r* < 0 stands for negative correlation. ^△^ *P* < 0.05 and |*r* | > 0.4 indicates the significant correlation between the two indexes.
